# Implementation of Cigarette Plain Packaging: Triadic Reactions of Consumers, State Officials, and Tobacco Companies—The Case of Saudi Arabia

**DOI:** 10.3390/ijerph17082668

**Published:** 2020-04-13

**Authors:** Marwah M. Hassounah, Abdulmohsen H. Al-Zalabani, Mohammed D. AlAhmari, Afraa A. Murriky, Anwar M. Makeen, Abdullah M.M. Alanazi

**Affiliations:** 1 Community Medicine Unit, Family and Community Medicine Department, King Saud University, Riyadh 11564, Saudi Arabia; marwah.m.i.h@gmail.com; 2 Department of Family and Community Medicine, College of Medicine, Taibah University, Al-Madinah 42353, Saudi Arabia; aalzalabani@gmail.com; 3 Department of Respiratory Care, Prince Sultan Military College of Health Sciences, Dhahran 34464, Saudi Arabia; m.alahmari@psmchs.edu.sa; 4 Department of Restorative Dentistry, Riyadh Elm University, Riyadh 11564, Saudi Arabia; afraa.muriky@riyadh.edu.sa; 5 Department of Family and Community Medicine, Jazan University, Jazan 88723, Saudi Arabia; amakeen@jazanu.edu.sa; 6 Department of Respiratory Therapy, King Saud bin Abdulaziz University For Health Sciences, Riyadh 11564, Saudi Arabia; 7 Sparkman Center for Global Health, The University of Alabama at Birmingham, Birmingham, AL 35205, USA

**Keywords:** communication, cigarettes, tobacco companies, plain packaging, standardized packaging

## Abstract

Objectives: In August 2019, Saudi Arabia started implementing plain packaging for cigarettes. Three months later, an opposing campaign on twitter using an Arabic hashtag “the new smoke” gained momentum amongst smokers. The purpose of this study is to document this opposing campaign’s timeline and describe consumers, government, and tobacco industry rhetoric. Methods: We created a timeline of the campaign events then performed online social listening of Arabic twitter hashtags related to the campaign. Results: Campaigners mainly complained of an unfavorable new taste in cigarette packs with plain packaging. The messaging developed to accusations to government entities and neighboring countries, and then after threats to boycott tobacco companies. The campaign received a significant amount of media coverage and elicited an official response from a number of Saudi government bodies, such as the Saudi Food and Drug Authority and Ministry of Commerce and Investment. Conclusion: This case points at a need for risk communication training, possible tobacco industry manipulation, and a need to gain consumer trust with evidence-based messaging techniques. The case of cigarette plain packaging adoption in Saudi Arabia serves as an example to other countries of potential consumer interaction, tobacco industry interference, and state official counter-reactions.

## 1. Introduction

In response to the global tobacco epidemic, the World Health Organization (WHO) introduced a Framework Convention on Tobacco Control (FCTC) to support governments in reducing tobacco demand and supply issues [1]. The FCTC is a progressive treaty that encourages the implementation of various articles including 11 and 13 [2,3]. These articles regulate the display of tobacco products in terms of adopting plain packaging (dark-brown simple packaging) [4] with a large pictorial health-warning label (HWL) [5], and restrictions to tobacco advertising and sponsorship, except for brand trademarks [3].

The effects of the implementation of the FCTC encouraged various countries including Australia, the UK, and France to adopt plain packaging for tobacco products [6,7,8]. The growing body of research reveals that plain packaging decreases tobacco use and sales, as well as government health costs [9,10,11]. The brown-black package has also been shown to lessen the appeal to use tobacco products and increase the noticeability and significance of the HWL [12,13,14]. For instance, the sales of tobacco products in Australia have declined by around 7.5% and the fear of smoking consequences has increased among French adolescents (prevalence ratio = 1.06, 95% confidence interval [1.02 to 1.09]) after the implementation of plain packaging [15,16]. Despite the promising outcomes of plain packaging, its diffusion has been slow because of the opposition of the tobacco industry and its attempts to impede tobacco control proposals [11,17].

Tobacco companies have a long history of lobbying against tobacco control initiatives to protect their trademarks and sales [11,17]. The main arguments against plain packaging center around violations of international treaties of intellectual property and tobacco trade, lack of sufficient evidence of plain packaging effects, and risk of black-market sales of illicit tobacco products [18,19,20,21,22,23,24]. Nevertheless, the adoption of plain packaging is increasing, regardless of tobacco industry campaigns and efforts to persuade the public [25].

In 2014, Saudi Arabia issued an anti-smoking law that reinforced previously implemented regulations, such as banning smoking in public and private sector workplaces, tobacco advertisement, selling to those younger than 18 years old, among other executive regulations [26]. In June 2017, a 100% taxation was imposed on tobacco products in Saudi Arabia with an increase in standard product price [27]. No population-level studies have measured the effect of taxation on smoking in Saudi Arabia; however, the only study done in the city of Jeddah shows that almost 40% of respondents were resistant to smoking cessation despite the taxation, and 29% changed to a cheaper brand [28].

In August 2019, the plain packaging policy took effect in the Saudi Arabian market to restrict the use and sale of cigarette tobacco products. As such, the country became the first to do so in the Middle East and North Africa (MENA) region [29]. Aligned with its 2030 vision for public health promotion, Saudi Arabia is determined to reduce the burden of tobacco consumption after the national prevalence of cigarette smoking increased from 12.2% in 2013 to 21.4% in 2018 [30,31]. In addition to other tobacco treatment and prevention services, the adoption of plain packaging is expected to discourage youth from smoking and help smokers to quit.

The purpose of this study is to document the case of the campaign opposing the implementation of cigarette plain packaging in Saudi Arabia. Here, we report the societal and sectoral reactions from consumers, state officials, health advocates, the media, and tobacco companies observed after increased complaints were tweeted on Twitter.

## 2. Materials and Methods

First, we established a timeline of events from professional observation and public announcements. Second, online social listening was used to assess and analyze the reactions to the implementation of plain packaging in Saudi Arabia. Arabic Twitter hashtags “the new smoke”, “the new smoke is adulterated”, “all types of tobacco kill”, and “tobacco war in the picture” were assessed from 19 November to 30 December, 2019. The purpose was to analyze the reactions and counter-reactions of the public, tobacco companies, health advocates, the media, and state officials to the “new smoke” campaign. Our findings are reported in the form of a timeline and narrative text. Third, we employed the Google Trend tool (trends.google.com) to assess Google searches on the Arabic equivalents of “tobacco,” “the new smoke,” and “adulterated smoke”. The trends emerging for these search terms in Saudi Arabia from 7 October to 30 December, 2019 are reported in a comparison graph.

## 3. Results

The following describes the cigarettes’ path from production to the consumer in Saudi Arabia, the key media events in this case, and sentiments of the Saudi smokers’ community participating in the examined Twitter hashtags. Table 1 shows a timeline of these events.

### 3.1. Tobacco Control and Sales in Saudi Arabia

In Saudi Arabia, inter-institutional collaborations operate to control tobacco, treat smokers, and prevent the youth from starting to smoke [32]. Major local vendors in Saudi Arabia (e.g., Al-Babtain Group, Al Nakhla Tobacco, Golden Leaf Tobacco, Villiger Söhne, Yousuf MA Naghi, and Sons’ Cigalah Group) [33] are obliged to adhere to numerous steps before selling their products in the Saudi market. To import tobacco products from international tobacco companies (e.g., British American Tobacco, Imperial Tobacco, Phillip Morris International) [33], vendors must request tax stamps for these products (a stamp for each tobacco pack) from the General Authority of Zakat and Tax (GAZT) [34]. After processing, GAZT ships the tax stamps to the international tobacco companies and collects tobacco tax. Tobacco companies are obligated to adhere to the national guidelines for tobacco plain packaging from the Saudi Food and Drug Authority (SFDA) [29] and to display the tax stamps issued by GAZT [34].

Saudi Customs then clears shipments to local vendors after the SFDA’s approval. The SFDA tests random samples of the imported tobacco products to ensure compliance with local tobacco packaging and content guidelines [29]. While local vendors distribute tobacco products to sales markets, three national entities control and prevent tobacco use inside the country. First, the National Committee for Tobacco Control monitors national tobacco use and issues tobacco control guidelines. Second, the Smoking Control Program at the Ministry of Health and Coordinating Committee for Anti-Smoking Associations (NGOs sector) provide programs to prevent smoking and tobacco treatment services.

### 3.2. Lobbying Against Plain Packaging

**Figure 1:**
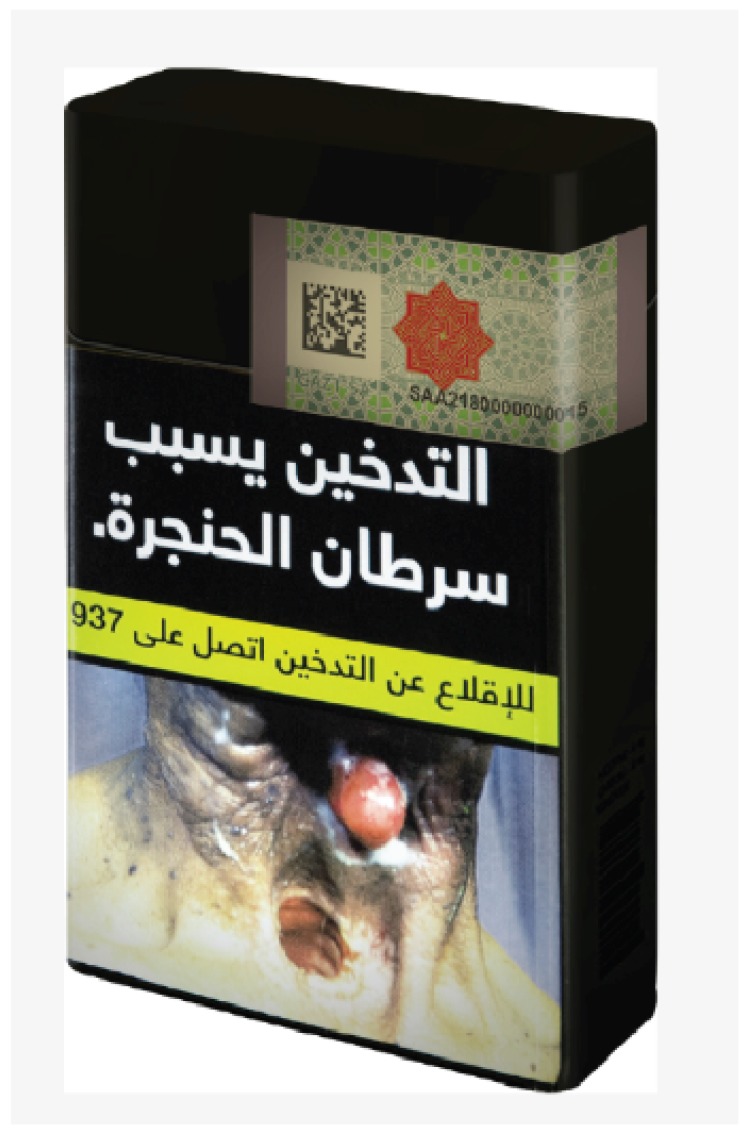
Sample of cigarette plain packaging in Saudi Arabia (GAZT, 2019). The sample description from the top: Tax Stamp, Health Warning Label, Quitline Phone Number, Large Pictorial Warning.

In August 2019, the plain packaging (Figure 1) regulation took effect. On 23 October 2019, the first tweet was posted pertaining to plain packaging using the hashtag “the new smoke” [35,36]. On 19 November, another hashtag was created (“the new smoke is adulterated”), which added to the hashtag the number of days passed since its launch; for example, “the new smoke is adulterated_14” [37]. By 26 and 29 November, 2019, “the new smoke” and “new smoke are adulterated” hashtags had gained traction, respectively [37,38]. The hashtags started with smokers’ complaints regarding the taste of the cigarettes in the packs with the new plain packaging. The tweets began distinguishing between the old and new cigarettes, implying a change in cigarette ingredients and quality. This was accompanied by accusing government entities for the change. Consistent themes and messages throughout the campaign included complaints of the taste, “bring the old cigarettes back,” videos displaying the characteristics of the “new smoke” and its differences from the old one, frustration with overpaying tobacco taxes and having their pleasure manipulated, and claims that smokers were being admitted to hospital after complications attributed to the ingredients in the new cigarettes.

**Figure 2:**
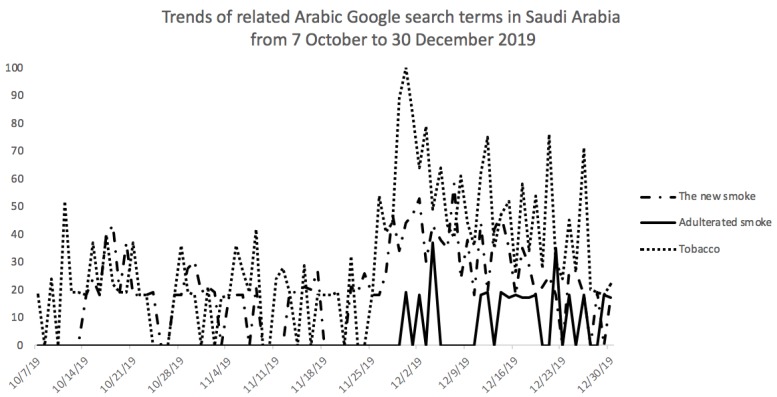
The numbers in the vertical axis represent search interest. A value of 100 is the peak popularity for the search term and a value of 0 means that there was not enough data for the search term (data accessed 6 January, 2020).

With more voices claiming the cigarettes were adulterated, the GAZT responded on 20 November, 2019 through its Twitter account. It provided images explaining the presence of taxation stamps on cigarette packs as proof of legality [39]. Hashtag users called on each other to file complaints with the Ministry of Commerce and Investment (MCI) for commercial fraud because the expiry dates were not included on the packets. On its Twitter account, the MCI stated that the issue was not under its jurisdiction, and deleted the tweet a few days later. Following this, the Ministry of Health Tobacco Control Program (MoH-TCP) received hundreds of emails and calls over a period of three days on the issue of the new cigarettes. There was also a significant increase in Google searches on the topic (Figure 2).

### 3.3. Officials Respond

In the absence of an official government statement, “fake” information started to emerge. A fake Ministry of Health (MoH) hazard warning circulated on social media, eliciting the first response from the MoH-TCP on 29 November, 2019 denying it had released the warning [40]. The next day, the SFDA released an official written statement on the issue [41]. The tweeted statement was entitled: “A clarification from SFDA on all new tobacco products being free of adulterated content”. The statement assured consumers that cigarettes are tested at points of entry, and none of the screening tests showed deviance from standard amounts of nicotine, carbon dioxide, tar, humidity percentage, or presence of wood sawdust as mentioned on Twitter. The same statement reiterated that plain packaging regulations do not ask manufacturers to change the ingredients, and that even if cigarettes comply with the standards, they are still harmful to health. To encourage checking for the origin of packets and their legality, the SFDA highlighted the GAZT taxation sticker and the mobile app “tahqaq,” which means “to check and confirm” [42].

Following this statement, many social media influencers tweeted in support of the SFDA, which led some hashtag users to express their skepticism regarding whether the SFDA had hired them. In addition, many tweets started promoting the purchase of cigarettes from other countries either online or over the border. At the same time, emerging English tweets tagging tobacco industry accounts demanded action, claiming that a change in taste and ingredients is harmful to company brands. To this, the British American Tobacco - Middle East Company (manufacturing Dunhill, Pall Mall, JPGL, Rothmans, Vogue, and Kent cigarettes) responded in an official statement [43] in which they confirmed adhering to Saudi Arabia’s plain packaging policy and denied any change to their products’ taste or tobacco mixture.

On the other hand, a tobacco control advocacy group started the counter hashtag “all types of tobacco kill” on Twitter, which started trending. The counter hashtag focused on the fact that all tobacco is harmful, provided a smoking cessation hotline, highlighted the religious aspect of smoking, noted tobacco industry manipulation, and provided evidence for the effectiveness of plain packaging. Known public figures in tobacco control efforts, such as the Secretary General of the National Tobacco Control Committee, as well as smoking cessation non-profit organizations, such as Purity, Aman, Safaa, Tdark, and Kafa, all tweeted the counter hashtag. The World Health Organization Regional Office for the Eastern Mediterranean (WHO EMRO) also tweeted in support of Saudi Arabia’s plain packaging policy [44], recognizing that the country was facing an agonistic campaign for its decision regarding plain packaging. Simultaneously, the MoH-TCP weekly statistics showed a 100%–700% increase in visits to smoking cessation clinics across the kingdom.

### 3.4. Opening a Public Investigation

Over the period 11–15 December, 2019, three government entities released statements, the most prominent being the MCI and SFDA’s joint investigation statement [45]. In the statement, they announced their call on all cigarette importation companies, their agents, and representatives in Saudi Arabia demanding explanations for consumers’ complaints and comments on the change in taste. The companies confirmed that they changed only the packaging according to the WHO’s plain packaging standards and as per Saudi Arabia’s new regulation.

As consumer input persisted, the SFDA and MCI deemed this response insufficient. They asked the companies to (1) disclose all cigarette contents before and after plain packaging was implemented; (2) provide the ingredients of concentrations and emissions, source of the tobacco, cigarette paper, and filters used, and where they were manufactured and assembled; (3) clarify reasons for the change in taste; and (4) perform taste tests and announce the results to consumers by the middle of the next week (meaning around 18 December 2019).

The joint investigative statement also mentioned that the SFDA sent seven samples of locally available tobacco types to an international laboratory (Eurofins.com) to study its quality and taste over the past two years. The results would be shared with the public as soon as the SFDA received them. Should the results indicate alterations, the SFDA and MCI would apply harsh sanctions to protect consumers. The Consumer Protection Association (CPA) issued a statement [46], demanded explanations from tobacco companies, and asked them to answer consumers’ inquiries directly. It supported the implementation of harsh and timely sanctions in the case of any confirmed manipulation and emphasized consumers’ right to comply with standards for registered products.

### 3.5. Media Coverage of The “New Smoke” Campaign

The campaign attracted the media’s attention, which increased coverage on smoking cessation and awareness topics. This included traditional media like television (Ya Hala Show, Tafa’olcom, Tarek Show, Al Ekhbariya), radio, and newspapers (Al-Watan, Okaz, AlRiya dh, Makkah, Al-Yaum), and new media, such as social media (the hashtags “thank you tobacco combaters” and “their experiences in smoking cessation and beyond”) and online news outlets (BBC.com, Alarabiya.com, eremnews.com, and Sabq.com) [47,48,49,50,51,52,53,54,55,56].

Here, we highlight a significant episode from a TV show named Fissora (in the picture) aired on 17 December 2019 for 1 h 20 min on the Rotana Khalijia channel [57]. It is significant because of the popularity of the program host and the group of policy makers, experts, and opinion makers hosted. The episode hosted the CEO of the SFDA, an Australian professor in health policy, the chairman of the governmental tobacco advisory committee when plain packaging was implemented in 2012, the governor of GAZT, and the governor of Saudi Customs, as well as Saudi marketing, legal, economic, and health experts. The interviews addressed the change in taste, adulterated content, tobacco companies’ response to plain packaging, and the black market for cigarettes. The economic expert was also a smoker and provided insight from a smoker’s perspective. Furthermore, cigarette suppliers were asked for comment, and they commented that smokers’ complaints were psychological.

Before airing the episode, hashtag users were concerned it would lecture on the harmfulness of smoking. After airing, sentiments toward the episode were generally negative, especially those regarding the government officials. Smokers sensed a contradiction and expressed their mistrust. The expert who smoked was very popular among hashtag users, who believed he represented them well. Consequently, they shared two phrases he mentioned in the episode. The first is: “This is our pleasure, if you play with our pleasure then bear with what you will get.” The second is: “I do not buy your laboratories. The true laboratory is the smoker’s mouth” [57].

### 3.6. Twist to the Campaign Narrative

Hashtag users challenged the barcode number of the plain package packs, which starts with 629 in reference to the United Arab Emirates (UAE), a country bordering Saudi Arabia. Hashtag users expressed their outrage at the notion that tobacco companies were now manufacturing in the UAE in an area near Dubai, namely Jebel Ali, instead of in Europe. Jebel Ali is a trade free zone, meaning a geographical area where industries and manufacturers can perform their operations while being exempt from certain taxes and customs duties [58]. Sarcastic, angry tweets belittled the quality and standard of goods manufactured in Jebel Ali, including tobacco and other products imported into Saudi Arabia. The sarcasm then shifted to a call to boycott all products from the UAE. The Emirates Authority for Standardization and Metrology (EASM) responded with a statement alluding to the ongoing accusations without explicitly mentioning Saudi Arabia or tobacco products [59]. It emphasized its rigor in maintaining the standards of the UAE and Gulf countries in all industrial processes, and asked consumers to avoid circulating false information on social media without proof and to direct their complaints to the regulating authority for action. Over the following three days, the EASM tweeted their commitment to standard specifics, explaining that a product barcode does not denote the manufacturing country and correcting the rumor that the UAE exported lower-quality products than those sold in the country [60,61].

In response to the aforementioned digression and aired episode of Fissora, Philip Morris International (manufacturing Marlboro, Parliament, Chesterfield, and L&M cigarettes) released a statement on 20 December 2019 [35]. It denied manufacturing cigarettes in the UAE and explained that the barcode number indicates the office at which the barcode was registered. Furthermore, it confirmed that the cigarettes distributed in Saudi Arabia are manufactured in Poland, Germany, and Turkey. Finally, it assured consumers that the quality or taste of its cigarettes had not been changed after implementing plain packaging.

### 3.7. Announcing the Results

Between the release of the joint investigation statement of the SFDA and MCI (11 December 2019) and the announcement of the results of the tobacco industry taste tests and SFDA laboratory tests (25 days later), no follow-up statement was issued. During this time, rumors arose about the SFDA backing out of the plain packaging regulation and fabricating the results of their tests. In addition, hashtag users wondered about the results, and accused the government of trying to manipulate their hashtags to hide them. The hashtag continued trending, and the notably dominant English tweets tagged tobacco industry accounts demanding their help in reinstating the “old cigarettes” or face consumer boycott.

On 4 January 2020, the SFDA retweeted the Saudi Press Agency (SPA) report of the results of the SFDA and independent international laboratory tests [62]. The results confirmed a change in some characteristics of the cigarettes that may have caused the reported change in taste. However, this change did not violate technical or standard specifications; it only affected user experience. Consequently, the investigating government taskforce directed tobacco companies to address this change; revert to the original taste; and to list on the plain packaging the ingredients, country of origin, and manufacturing date as soon as possible. Finally, the taskforce directed tobacco companies and their agents to directly communicate with consumers through dedicated call centers to handle complaints and comments, and resolve them under the supervision of the MCI.

## 4. Discussion

Smokers’ response to the implementation of plain packaging in Saudi Arabia was strong and loud on both social and traditional media channels. The response focused on the perceived poor taste of the new plain-packaged cigarettes. When implemented in Australia in 2012, smokers also reported the perceived poor taste of plain-packaged cigarettes [63]. However, based on market testing, tobacco companies have long known that packaging the same cigarettes in different packages affects smokers’ taste rating [64]. In addition, experimental studies have shown that branded cigarettes are rated as having a better taste than identical concealed cigarettes [65]. Moreover, population studies and the plain packaging of other tobacco products, such as cigars and roll-your-own cigarettes, were found to induce the same effects [65,66].

In addition to the perceived poor taste, plain-packaged cigarettes were considered lower quality, more harmful, and associated with less satisfaction. These perceptions were consistent with the results of previous studies investigating the effect of plain packaging on consumer perceptions and indicate a reduction in the appeal of cigarettes, which is one objective of plain packaging [67]. An increase in the effectiveness of health warnings, another objective of plain packaging, was also observed in the public comments.

Despite the evidence supporting the effect of packaging on perceived taste and quality, we cannot exclude possible manipulation by the tobacco industry. Two possibilities have emerged from our online social listening for this social media campaign: 1) tobacco companies altering the constituents of cigarettes to link the bad taste with the plain packaging policy, and 2) tobacco companies interfering with social media content to show more negative sentiment toward the new packaging. The first consumer reaction in social media started after two months of cigarette plain packaging implementation, which also suggests that the taste of the new cigarettes was changed by the tobacco companies after the policy implementation. Recognizing the impact on their brand’s appeal, the tobacco industry has fiercely resisted the implementation of plain packaging in many countries, utilizing several approaches, including legal challenges, lobbying, and public relations campaigns [68,69]. Other countries have employed the tactic of refocusing policy discussion on the claimed negative effects such as increased smuggling [68]. This was also evident in social media interactions in Saudi Arabia. The tobacco industry’s activities to undermine tobacco control efforts are known worldwide, including in the Gulf Cooperation Council (GCC) countries. Tobacco-related documents revealed the various strategies and approaches tobacco companies employ to counteract tobacco control in GCC countries [70].

Although there was a two-month gap between plain packaging implementation and the first consumer reaction in social media, the established timeline of events shows that consumer rhetoric started slowly and steadily before gaining collective momentum and attention until it reached a climax at the airing of the television show interview. The episode was the first opportunity to bring decision makers up front and ask them about the issue. Until that point, government entities had used Twitter as their main channel to convey official statements and information. The SFDA alone, being the main player in this case from the government side, has one million followers on its Arabic twitter account. It is worth mentioning that in 2019, 56% of internet users in Saudi Arabia were Twitter users, of which 71% were males averaging 26 years of age [24,28,71].

Government agencies’ response at the start of the public interaction is considered suboptimal. The agencies were slow in responding to hashtags and smokers’ concerns regarding plain packaging, and the first responses were mainly those of defense and denial. This initial response, combined with the lack of communication prior to implementing plain packaging, exacerbated smokers’ mistrust and provided room for speculation and accusation. When people distrust the authorities, they are unlikely to follow their advice or believe official statements. In the later stages, the agencies caught up and began to more carefully curate their communication messages. We believe this incident provides an opportunity for these government agencies to evaluate the effectiveness of their communication strategies, identify gaps, and formulate a strategic plan for communication in the future [72].

The Saudi case of cigarette plain packaging highlights lessons for the future adoption of any tobacco control policy. The period before policy implementation is a significant time for the evidence-based health communication needed to educate and engage the public. The pre-implementation period could be also utilized to set the scene for the announcement of plain packaging. That could be done by holding focus groups, exercising online social listening, and creating audience segmentation personas to have a better understanding of the target audience. This equips communication preparedness efforts to use the audience’s psychographic in wording, messaging approach, and preferred media and channels. In addition, this time could be utilized by state officials and health advocates from NGOs and government sectors to partner, anticipate, strategize, and build trust with consumers. After policy implementation, state officials and health advocates should accommodate possible consumer frustration and refusal through proactive, clear, consistent, and timely communication. Preventive steps should be taken when implementing plain packaging for other tobacco products (smokeless, cigar, water pipe) in the future to prevent similar incidents.

There is room for further progress in demonstrating the case of plain packaging in Saudi Arabia. Future structured, qualitative research should clarify smokers’ perception of the implementation of plain packaging. In addition, hashtags related to plain packaging on Twitter should be content analyzed to provide a comprehensive picture of consumer reaction and smoking experience in the public arena. Policy evaluation of the effectiveness of plain packaging is a pivotal future step to study the temporal effects of its diffusion and associated consequences. In other countries, we endorse the careful implementation of plain packaging to avoid conflict between consumers, state officials, and tobacco vendors and manufacturers. To this end, assessing smokers’ awareness of plain packaging and interference from the tobacco industry, as well as empowering state officials, could serve as factors driving the implementation of plain packaging.

There are several limitations to this study. The introduction of plain packaging was accompanied by establishing several smoking cessation centers and strong health-related warnings. Thus, the Twitter campaign cannot be exclusively related to this implementation. It is unclear if the implementation of plain packaging as a separate strategy would elicit the same reaction. Second, the Twitter case started three months after the introduction of plain packaging. Experts cannot explain the lag between its implementation and the start of the Twitter campaign. Third, we examined the Twitter campaign over a three-month period during the peak of the reaction crisis; however, this is a relatively short period. Fourth, we alluded to possible behavioral changes (increase in smoking cessation visits) accompanying the campaign; however, it is not possible to accurately relate these to one factor (promoting smoking cessation on Twitter at the time of the campaign, actual change in taste, or another factor). Finally, the incident cannot be generalized to other countries in which plain packaging is implemented.

## 5. Conclusions

Since tobacco companies have a solid strategy in responding to anti-tobacco policies and regulations, a well-structured communication strategy by the government for before and after policy implementation is crucial. A proactive communication approach with a clear and consistent narrative decreases the potential of rumors, mistrust, and resistance from the general population of smokers.

## Figures and Tables

**Table 1:** Plain packaging and counter campaign timeline.

Date	Actor					Event
	Government	Tobacco Industry	Media	Consumers	External Entity	
23 Aug 2019	x					SA started PP implementation
23 Oct 2019				x		First use of the Arabic new smoke hashtag on twitter in relevance to SA PP
20 Nov 2019	x					GAZT tweets about recognizing legal cigarette packs from taxation label
26 Nov 2019				x		Anti PP campaign on the Arabic *new smoke* hashtag is trending on twitter
29 Nov 2019				x		Increase in calls and hundreds of emails to the MoH-TCP pertaining to the “new smoke”
29 Nov 2019	x					MCI denies responsibility of change in cigarettes on twitter, then deletes tweet a few days later.
29 Nov 2019				x		Counter hashtag *all types of tobacco* trending on twitter
29 Nov 2019	x					MoH TCP disclaims a circulating fabricated MoH statement
30 Nov 2019	x					SFDA releases its official statement
5 Dec 2019					x	The WHO tweets on the anti PP campaign in support of SA government efforts
5 Dec 2019				x		100% to 700% increase in visits to MoH smoking cessation clinics across SA
6 Dec 2019		x				British American Tobacco Middle East releases its official statement denying change to cigarettes
11 Dec 2019	x					SFDA and MCI release a joint investigation statement
13 Dec 2019					x	EASM releases its official statement on complying to standards
15 Dec 2019	x					CPA releases its official statement supporting investigation of tobacco companies
17 Dec 2019			x			*Fissora*, a popular TV talk show, hosts an episode with key experts and decision makers on the new smoke controversy
20 Dec 2019		x				Philip Morris releases its official statement denying change to cigarettes or manufacturing country
4 Jan 2020	x					SFDA releases cigarette testing results

PP: plain packaging, WHO: the World Health Organization, SA: Saudi Arabia, MCI: Ministry of Commerce and Investment, SFDA: Saudi Food & Drug Authority, MoH: Ministry of Health, TCP: tobacco control program, CPA: Consumer Protection Association, GAZT: General Authority of Zakat and Tax, EASM: Emirates Authority for Standardization and Metrology.
